# Spectroscopic, Thermally Induced, and Theoretical Features of Neonicotinoids’ Competition for Adsorption Sites on Y Zeolite

**DOI:** 10.3390/molecules30153267

**Published:** 2025-08-04

**Authors:** Bojana Nedić Vasiljević, Maja Milojević-Rakić, Maja Ranković, Anka Jevremović, Ljubiša Ignjatović, Nemanja Gavrilov, Snežana Uskoković-Marković, Aleksandra Janošević Ležaić, Hong Wang, Danica Bajuk-Bogdanović

**Affiliations:** 1 Faculty of Physical Chemistry, University of Belgrade, Studentski Trg 12–16, 11158 Belgrade, Serbia; bojana@ffh.bg.ac.rs (B.N.V.); maja.rankovic@ffh.bg.ac.rs (M.R.); anka@ffh.bg.ac.rs (A.J.); ljignjatovic@ffh.bg.ac.rs (L.I.); gavrilov@ffh.bg.ac.rs (N.G.); danabb@ffh.bg.ac.rs (D.B.-B.); 2 Faculty of Pharmacy, University of Belgrade, Vojvode Stepe 450, 11221 Belgrade, Serbia; snezana.uskokovic@pharmacy.bg.ac.rs (S.U.-M.); aleksandra.janosevic@pharmacy.bg.ac.rs (A.J.L.); 3 School of Chemical Engineering & Technology, China University of Mining and Technology, Xuzhou 221116, China; hongwang@cumt.edu.cn

**Keywords:** neonicotinoids, Y zeolite, competitive adsorption, DFT/semiempirical modeling, environmental remediation

## Abstract

The competitive retention of pollutants in water tables determines their environmental fate and guides routes for their removal. To distinguish the fine differences in competitive binding at zeolite adsorption centers, a group of neonicotinoid pesticides is compared, relying on theoretical (energy of adsorption, orientation, charge distribution) and experimental (spectroscopic and thermogravimetric) analyses for quick, inexpensive, and reliable screening. The MOPAC/QuantumEspresso platform was used for theoretical calculation, indicating close adsorption energy values for acetamiprid and imidacloprid (−2.2 eV), with thiamethoxam having a lower binding energy of −1.7 eV. FTIR analysis confirmed hydrogen bonding, among different dipole-dipole interactions, as the dominant adsorption mechanism. Due to their comparable binding energies, when the mixture of all three pesticides is examined, comparative adsorption capacities are evident at low concentrations, owing to the excellent adsorption performance of the FAU zeotype. At higher concentrations, competition for adsorption centers occurs, with the expected thiamethoxam binding being diminished due to the lower bonding energy. The catalytic impact of zeolite on the thermal degradation of pesticides is evidenced through TG analysis, confirming the adsorption capacities found by UV/VIS and HPLC/UV measurements. Detailed analysis of spectroscopic results in conjunction with theoretical calculation, thermal profiles, and UV detection offers a comprehensive understanding of neonicotinoids’ adsorption and can help with the design of future adsorbents.

## 1. Introduction

Neonicotinoids (NNs) are systemic insecticides that act as agonists of nicotinic acetylcholine receptors in the insect nervous system. By overstimulating these receptors, NNs disrupt normal neural transmission, leading to paralysis and mortality. Due to their high selectivity for insects over mammals, they have been widely used in agriculture for pest control [1,2,3]. However, NNs act indiscriminately, with a risk that non-target organisms and the surrounding soil and water will be adversely affected. In that way, their use in agricultural and urbanized areas has led to the contamination of non-target ecosystems. Despite bans on certain neonicotinoids in agriculture in some parts of the world, their continued use in biocidal products and public health initiatives remains a global issue. It is important to point out that neonicotinoids undergo biotic and abiotic processes producing products that can be more toxic than the parent compounds [4]. Pesticide formulations, which combine active ingredients with adjuvants, often exhibit greater toxicity to non-target species than the active compounds alone [5]. This increased toxicity is frequently attributed to the presence of adjuvants in the formulations. Therefore, it is crucial to study the adsorption behaviors of both pesticide formulations and their active ingredients to assess their environmental fate and potential risks [6,7,8,9].

Different methods may be employed for the removal of these pollutants [10,11]. However, researchers often investigate adsorption due to the variety of representatives and their derivatives present in the environment [12,13]. This commonly used physical remediation method enables nonselective pollutant retention with a high capacity, efficiency, and applicability on a large scale. Therefore, it is essential to comprehend the phenomenon of NN adsorption thoroughly. Some of the most common NNs in commercial insecticide products are acetamiprid (AA), imidacloprid (IC), and thiamethoxam (TM), either individually or in mixtures. Although many works deal with the adsorption of pesticides as a method of water remediation, the removal of individual pesticides is the main focus.

Advancing material science has produced various adsorbents, which have been investigated for AA in the literature [14]. Activated carbon prepared from pistachio shells and its modification by FeCl_3_ has shown a maximum adsorption capacity of around 85 mg g^−1^ [15]. Significantly lower adsorption capacities, calculated from the Langmuir monolayer as 32, 15, and 5 mg g^−1^ for methomyl, imidacloprid, and acetamiprid, respectively, were obtained for wood-chip biochar [16]. Panić et al. evaluated the effects of the initial concentration of TM solution in water, temperature, pH, adsorbent loading, and contact time on TM adsorption efficiency using oxidized, multi-walled carbon nanotubes as adsorbents. A fractional factorial design was employed, and a maximum adsorbed amount of 59 mg g^−1^ was achieved under the optimal conditions [17]. The work of Liu et al. investigated IC adsorption kinetics and isotherm models on polar microplastics under environmental conditions before and after photoaging [18].

Due to the multiple targets of NNs and the complexity of matrices, their detection typically requires chromatography-based separation methods. Liquid chromatography is well-suited for separating neonicotinoids due to their high polarity and low volatility. A diode array detector is frequently used for their detection, as NNs exhibit characteristic ultraviolet spectra [19]. As a simple and widely available technique, ultraviolet–visible spectroscopy is fast and sensitive for detecting these components. Therefore, even with no advanced separation and detection methods, UV absorption enables a straightforward preliminary quantification of neonicotinoids. A complete image of NNs detected in polluted waters and their fate is made up of the results acquired using different methods and extended views of different interactions in the environment. Different analysis techniques may be applied to assist in the resolution of NNs’ interaction with solid surfaces [20]. For instance, HPLC–UV was used to test acetamiprid, imidacloprid, thiacloprid, and thiamethoxam from river water and peanut milk samples when magnetic-ordered porous carbon/ZSM-5 was used for the extraction. The calibration curves for the four insecticides in the water sample showed linearity up to 200 ng mL^−1^ for selected NNs [21]. Another interesting technique, infrared difference spectroscopy, was considered, dealing with IC metabolites’ competitive binding with imidacloprid on Ca-montmorillonite and humic acids [22]. IC was moderately adsorbed, with the distribution coefficient of IC on HA being substantially greater than that on the Ca-clay. The sorption of IC was reduced by imidacloprid-urea in both Ca-clay and humic acids, indicating the occurrence of competitive sorption between these two compounds. This points to the possible binding of the ring -NH- group, which remains unaffected by urea product formation. However, the work by Liu et al. on quantitative surface and Hirshfeld surface analyses of AA showed that nitrogens from the single-bonded CN group and the six-membered ring can act as hydrogen-bond acceptors, forming N⋯H-O interactions with different polar solvents. Positive potential regions were detected near the hydrogen atoms in the single-bonded CH_3_ and >CH_2_ groups, but without acidic hydrogen atoms, they cannot function as Lewis acids in solution [23]. This leaves only protonated nitrogens of IC to act as proton donor sites, while AA acts as a proton acceptor of bridging hydroxyls in a zeolite structure, as proposed in our previous work [24]. Infrared spectroscopic analysis with simultaneous testing of pesticide removal and antioxidant activities resolved the nature of the interaction. Redox processes can be efficiently used to address pesticide removal, such as simultaneous degradation of IC, TM, and dinotefuran in soil by a nanoscale zero-valent Fe-activated persulfate system [25]. SPE cartridges packed with thioether MOF were used for the removal of NN mixtures containing thiamethoxam, clothianidin, imidacloprid, acetamiprid, and thiacloprid at different concentrations [26]. However, a comprehensive description of simultaneous adsorption from aqueous solutions is still missing.

**Figure 1:**
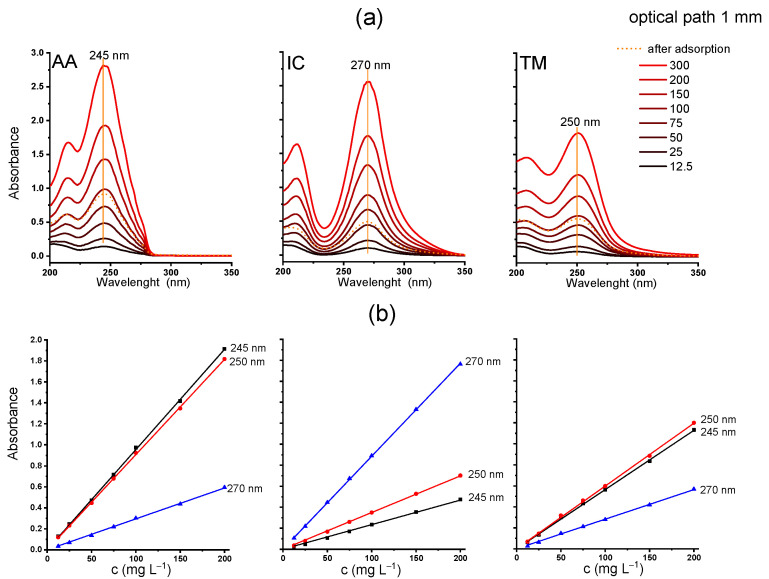
(**a**) UV spectra of acetamiprid (AA), imidacloprid (IC), and thiamethoxam (TM) at different concentrations (300, 200, 150, 100, 75, 50, 25, and 12.5 mg L^−1^), with the spectra of the supernatant from AA, IC, and TM/zeolite suspension (initial NN concentration 300 mg L^−1^); (**b**) calibration curves at λ_max_ (245, 270, and 250 nm); optical path: 1 mm.

In addition to the methods explored in the description of complex systems and removal options, materials design is a key element, whether they are used as adsorbents or catalysts [27]. A recent review presented a systematic and comparative overview of the application of mesoporous silica materials and synthetic zeolites and showed that they are effective for a wide range of pesticides [28]. We tested MFI, FAU, and BEA zeolite frameworks with different Si/Al ratios for AA and IC adsorption, and the best results were detected for the FAU zeotype for samples with Si/Al ≥30, or over 200 mg g^−1^. FAU contains the most isolated proton donor centers accessible to adsorbate molecules among the investigated zeolites [24]. Alongside this, the FAU framework is thermally stable, which enables thermal treatment as an efficient regeneration method. Thermal treatment at 300 °C enables rapid and low-energy-demanding recovery procedures with 65% efficacy. Moreover, heat treatment at 700 °C completely recovers the initial efficacy after several low-temperature regenerations [10]. As an important aspect is the reuse and/or recycling of spent adsorbents, the thermal treatment of acetamiprid-saturated Y zeolite at 700 °C under argon was recently investigated [29] as a way to produce nitrogen-rich carbon materials for use in supercapacitors. In the present study, three representative NNs, AA, IC, and TM, were selected as target pollutants (Figure 1) for Y zeolite adsorption studies. The simultaneous adsorption of AA, IC, and TM from the aqueous solution by Y zeolite was investigated using UV spectrophotometry, HPLC/UV detection, and TG analysis. FTIR spectroscopy and DFT calculations produced a comprehensive picture of overall interactions, coadsorption tendencies, and bonding mechanisms.

## 2. Results

### 2.1. Experimental Investigation of the Adsorption of Individual NNs on Y Zeolite

The UV spectra of the three NN solutions of different concentrations were measured in the spectral range of 200–350 nm (Figure 1a). To analyze NN calibration curves, the least-squares linear regression analysis was carried out, and the results are shown in Figure 1b. An excellent linear correlation was obtained when cuvettes with an optical path of 1 mm were used, with the Pearson’s coefficients of 0.99995, 0.99998, and 0.99989 for AA, IC, and TM at the maximum absorption, i.e., 245, 270, and 250 nm, respectively (Table 1). Based on the slope of the calibration curve, the molar absorption coefficients ε at the wavelengths of maximum absorption for individual NNs were determined. Their ε values are similar, from 2265 m^2^ mol^−1^ for IC, then 2127 m^2^ mol^−1^ for AA, to 1744 m^2^ mol^−1^ for TM. The obtained results agree with the values reported in the literature, i.e., 2205, 1970, and 1680 m^2^ mol^−1^ for IC, AA, and TM, respectively [30]. When measuring concentrated solutions, cuvettes with shorter optical path lengths are practical, while for more diluted solutions, cuvettes with longer path lengths are necessary to ensure optimal absorbance readings. The spectra and data obtained using 10 mm cuvettes are shown in Figure S1 and Table S1. It is evident that the amount of individual NN adsorbed on Y zeolite can be reliably determined based on UV/Vis spectroscopy, with the limit of detection (LOD) at 3–5 mg L^−1^ and limit of quantification (LOQ) at 5–13 mg L^−1^ when an optical path of 1 mm is used. Notably, these limits are lower when thicker cuvettes are used, as estimated from the calibration curve and standard deviations [31]. The calculated LOD and LOQ values, using cuvettes with optical path lengths of 1 mm and 10 mm, are provided in Table 1 and Table S1, respectively.

**Figure 2:**
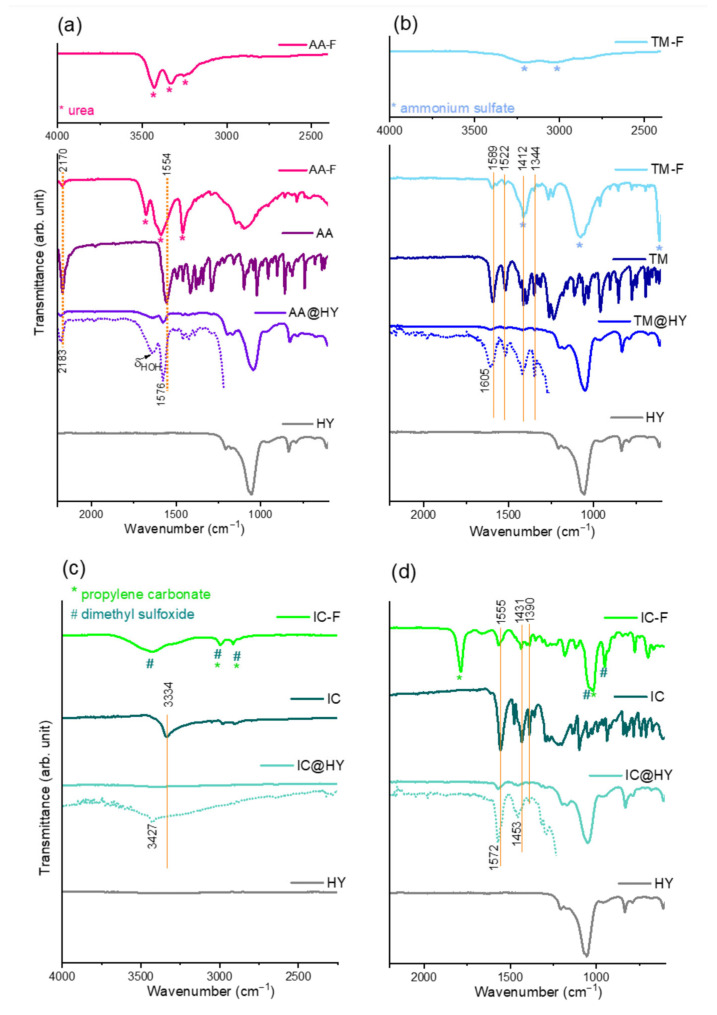
(**a**) FTIR spectra of NN formulations (F), (**b**) NN of technical purity, (**c**) NN adsorbed on HY zeolite (@HY), and (**d**) pure HY zeolite. The spectra represented by dotted lines have an expanded *Y*-axis for clarity.

The shapes of absorption bands of AA, IC, and TM after a 24 h contact time with adsorbent remain unchanged (dashed line in Figure 2a), and based on the reduced intensity of the bands, the adsorbed amounts were determined as 204, 244, and 207 mg g^−1^, i.e., 0.92, 0.95, and 0.71 mmol g^−1^, for AA, IC, and TM, respectively. HPLC measurements yielded similar results, confirming the excellent adsorption capacities of HY zeolites for all the tested NNs: 206.9, 237.8, and 199.2 mg g^−1^, i.e., 0.93, 0.93, and 0.68 mmol g^−1^ for AA, IC, and TM, respectively. On average, 215 mg g^−1^ of each NN is adsorbed when the initial solution concentration of the NN solution is 300 mg L^−1^ and the dose is 1 mg mL^−1^ for 24 h. The resolution of chromatogram peaks remains unaffected by the adsorption test and follows the linear calibration parameters, as shown in Figure S2 and Table S2.

However, when commercial pesticide formulations are used, the values of adsorbed NNs are significantly lower, 174, 146, and 165 mg g^−1^ (i.e., 0.79, 0.57, and 0.57 mmol g^−1^), for AA, IC, and TM, respectively (the concentrations of NN in the formulation, both before and after adsorption, were determined using the calibration curves shown in Figure 2). Corresponding results were obtained based on HPLC measurements: AA 167.8, IC 144.9, and TM 170.0 mg g^−1^.

The reason for the reduction in adsorption capacities lies in the various adjuvants made in commercial formulations. Based on the FTIR spectra shown in Figure 2, it is evident that urea and ammonium sulfate were used in the powdered formulations, AA and TM, respectively, while the liquid formulation IC includes solvents, propylene carbonate, and dimethyl sulfoxide. The adjuvants’ vibrational bands are marked with asterisks in the corresponding spectra. Due to the differences in adjuvant composition, the most pronounced drop in adsorption capacity (about 40%) was observed for the IC formulation. Ammonia- and amide-containing adjuvants (present in TM and AA) enhance binding to zeolite centers, whereas organic solvents in the IC formulation hinder adsorption in aqueous suspensions.

**Figure 3:**
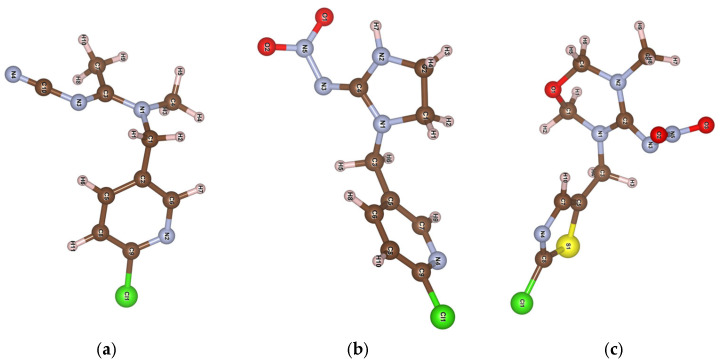
MOPAC software optimized structure with the atomic numbering of (**a**) acetamiprid, (**b**) imidacloprid, and (**c**) thiamethoxam.

The different adsorption of certain NNs may be influenced by their varying solubility in water. Reported findings for competitive adsorption of nitrophenol compounds on FAU zeolites [32] reveal that lower solubility leads to a greater extent of adsorption. Here, nitro-containing TM and IC show adsorption behavior inversely proportional to their solubilities (TM 4.1 g L^−1^ and IC 0.6 g L^−1^). An interesting proposition on thioether-based MOFs for adsorption of neonicotinoid series shows that acetamiprid is completely removed in the 0.1–100 mg L^−1^ range, while IC and TM sustain significant removal only at a 0.1 mg L^−1^ concentration [26]. Unlike the thioether-based MOF, the HY zeolite proved almost equally efficient for all three tested NNs. Although all the tested insecticides have similar basic structures, slight differences in the position and type of functional groups can significantly affect their interaction with the adsorbent. Figure 3 presents the MOPAC2016 open source software (Stewart, J.J.P., Stewart Computational Chemistry: Colorado Springs, CO, USA, 2016)-optimized structures of the examined NNs. Namely, imidacloprid and thiamethoxam are nitroguanidine-type neonicotinoids characterized by N-nitro (–N–NO_2_) group structures that contain oxygen atoms. In contrast, acetamiprid belongs to the cyanoamidine class, characterized by the N-cyano (=N−C≡N) group without oxygen atoms in its structure [33].

The influence of functional groups on adsorption can be monitored by comparison of the NNs’ FTIR spectra before and after adsorption. In the spectra of zeolite saturated with individual NNs, AA@HY, IC@HY, and TM@HY (Figure 2), asymmetric stretching vibration of internal tetrahedra, double ring modes of external linkages, and bending vibration in internal tetrahedra of the HY zeolite are observed, according to the Flaningen classification [34].

In the AA spectrum, many bands are found in the fingerprint region (below 1500 cm^−1^), but the most intense bands are centered at 2170 and 1554 cm^−1^, corresponding to C≡N and C=N vibrations, respectively. These bands are free from overlapping with the HY bands, making them very important for monitoring potential interactions during adsorption. Upon adsorption, these AA bands shift to higher wavenumbers, appearing at 2183 and 1576 cm^−1^ in the AA@HY spectrum, indicating significant interaction between AA and the zeolite surface [10]. Previous studies have reported that IR spectra obtained using the fluorolube mull technique reveal the absence of the isolated hydroxyl group band at 3630 cm^−1^ after acetic acid adsorption on zeolite [24]. Thus, hydrogen bonding is established between AA nitrogens and the bridging hydroxyl groups of the zeolite.

Similar to AA, the TM spectrum consists of a large number of bands, but the most intense bands in the region (not overlapping with the HY bands) can be selected for tracking interactions. These bands are attributed to the vibrations of characteristic functional groups. Specifically, the band at 1589 cm^−1^ corresponds to C=N stretching vibration, while the bands at 1522 cm^−1^ and 154 cm^−1^ are attributed to asymmetric and symmetric NO_2_ stretching vibrations, respectively. Additionally, the band at 1412 cm^−1^ is linked to C–H bending vibration [35]. In the case of TM adsorbed onto the zeolite, the C=N stretching vibration band shifts to 1605 cm^−1^, but changes are also evident in the region of vibrations centered around 1410 cm^−1^.

In the IR spectrum of pure IC, the absorption band observed at 3334 cm^−1^ corresponds to the N–H stretching vibration. The strong band at 1555 cm^−1^ is assigned to the NO_2_ asymmetric stretch of the nitro group and C=N stretching in the imidazoline ring. Additionally, the band at 1390 cm^−1^ corresponds to the NO_2_ asymmetric stretch, while the band at 1431 cm^−1^ is attributed to the CH_2_ scissoring modes. However, when IC is adsorbed onto the zeolite, this N–H vibration band significantly shifts toward higher wavenumbers. At the same time, the peaks at 1555 and 1431 cm^−1^ undergo much less notable changes, suggesting that NH/O hydrogen bonding with the zeolite surface is the predominant interaction mechanism between IC and zeolite [24,36].

### 2.2. Theoretical Modeling of Interactions

**Figure 4:**
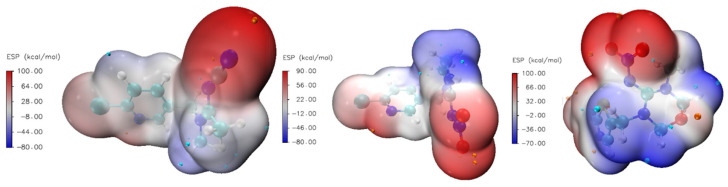
Electrostatic surface potential (ESP) maps for AA, IC, and TM.

García-Hernández et al. [37] calculated that electrostatic and dispersion interactions dominate adsorption of neonicotinoid on microplastics, while Shi et al. put forward π-π electron donor–acceptor interactions and H-bonding, covalent bonding, and hydrophobic interactions as dominant interactions on biochars [38]. This provided a quicker calculation of electrostatic surface potential (ESP) maps for the studied molecules (Figure 4).

**Figure 5:**
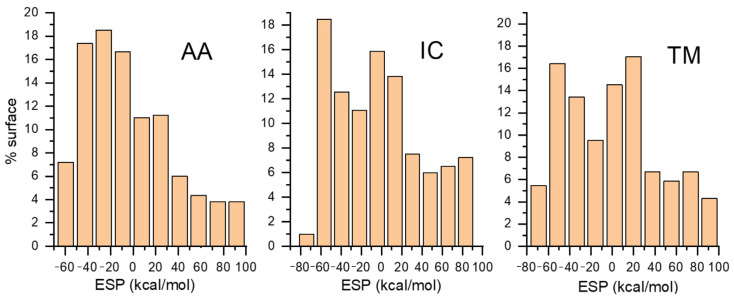
Percentage distribution of ESP surface as a function of ESP value.

These maps visualize the electrostatic potential distribution around a molecule, highlighting electron density and charge distribution regions, which are crucial for understanding molecular interactions, reactivity, and binding affinities. The color bar represents the strength range for ESP in kcal mol^−1^, which is very close in magnitude to ones calculated for similar systems [37,38]. The ESP range is almost identical for all three investigated molecules. Dots projected on the ESP surface represent extreme values of positive and negative parts. The red color represents electron-rich regions, while the blue color represents electron-poor regions. Electron-rich regions are around nitrogen and oxygen atoms, so we expect these parts to be included in interactions with the zeolite framework through hydrogen bond formation. The calculated percentage of the surface with a particular ESP value is shown in Figure 5. We can observe that a significant part of the molecule is the electron-poor region.

**Figure 6:**
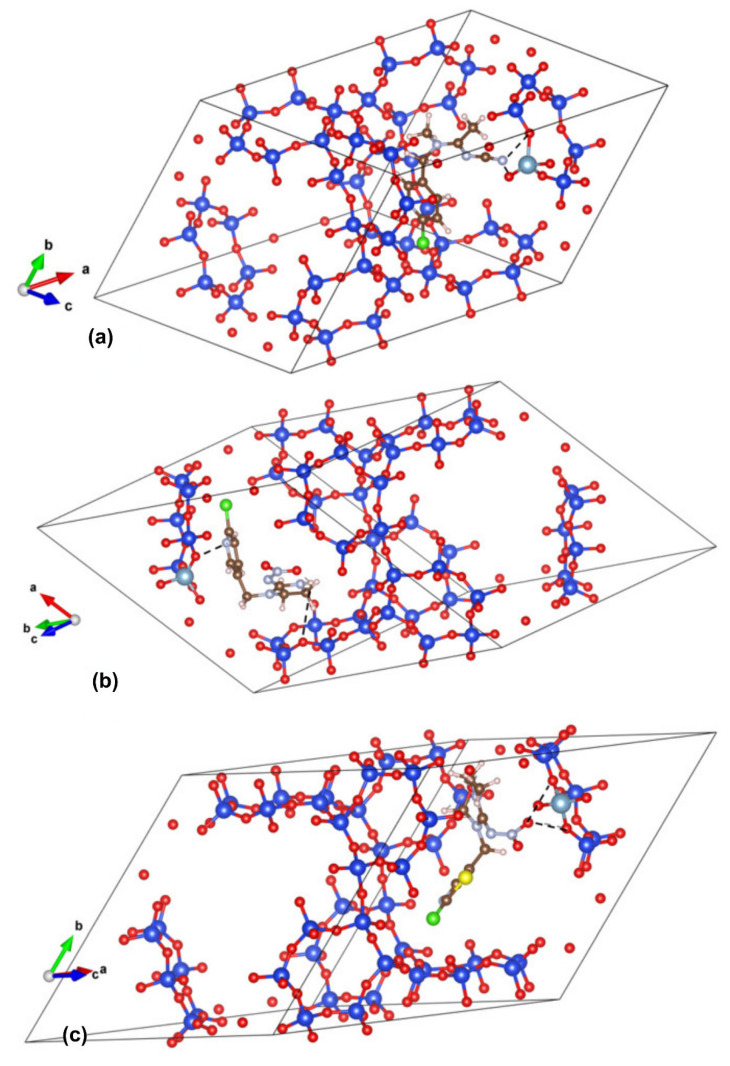
DFTB+ optimized interactions between (**a**) AA, (**b**) IC, and (**c**) TM and Y zeolite framework with zoomed-in sections on the right.

The calculated adsorption energies of −2.21 eV for IC and −2.18 eV for AA are comparable, while a less negative value of −1.70 eV for TM suggests a slightly weaker interaction. The optimized structures/interactions are presented in Figure 6. In AA, the charge distribution indicates that the primary binding sites are concentrated around the C≡N and C=N groups in full agreement with the findings of FTIR spectroscopy. For imidacloprid, FTIR spectra and previous antioxidant activity analyses indicate that IC acts as a proton donor [24], resulting in stronger interactions at the N-H site within the imidazoline ring. Calculations suggest that oxygens in the TM end group serve as anchor points to the zeolite framework via dipole–dipole interaction. The resulting effect is elongation/weakening of the adjacent C(12)-N(8) bond, seen in shifting its FTIR band to higher wavenumbers. Here, the lesser extent of TM interaction with zeolite surfaces in comparison with AA and IC is associated with its slightly less negative adsorption energy.

### 2.3. Investigation of Adsorption of NNs from the Mixtures on Y Zeolite

To examine the extent of chemical interactions between the investigated NNs when coexisting in the solution, we investigated whether the analytical response of the mixture coincided with the sum of the individual responses of the components. The spectra of the solution in which all three NNs are present in the same mass ratio, marked as NN300, NN180, etc., show that the asymmetric band with a maximum of around 250 nm fully corresponds to the mathematical sum of the spectra of individual NNs with the appropriate concentrations (Figure S3). Therefore, the examined NNs do not chemically interact at either low or high concentrations. The offset of the calculated and recorded spectral line below 220 nm arises from the tail of the UV absorption band of water and lower instrument sensitivity near the end of the range.

Since there is no interdependency between the absorbance of each component, Beer’s law can be applied, and the following equations for absorbance (A) at three different wavelengths can be considered:

ε_AA_^245^ b C_AA_ + ε_IC_^245^ b C_IC_ + ε_TM_^245^ b C_TM_ = A^245^ (1)

ε_AA_^250^ b C_AA_ + ε_IC_^250^ b C_IC_ + ε_TM_^250^ b C_TM_ = A^250^ (2)

ε_AA_^270^ b C_AA_ + ε_IC_^270^ b C_IC_ + ε_TM_^270^ b C_TM_ = A^270^ (3)

where ε_AA_, ε_IC_, and ε_TM_ are the molar absorptivities of AA, IC, and TM, b is the path length of the radiation through the sample, while C_AA_, C_IC_, and C_TM_ are concentrations (mol L^−1^) of the individual NN (to be determined). The molar absorptivity values, ε_AA_, ε_IC_, and ε_TM_, can be obtained from calibration curves for standards at the appropriate wavelengths (Figure 2b and Table 1). To examine the applicability of this method for the NN mixture, a series of solutions with known concentrations were prepared in a cumulative range of 12–300 mg L^−1^, with an equal proportion of each pesticide in the mixture. The concentrations, determined using the above equations and appropriate UV/Vis spectra, are presented in Figure S3. The results indicate that the method is suitable for estimating the concentration of each pesticide in a mixture within the range of 7–100 mg L^−1^.

**Figure 7:**
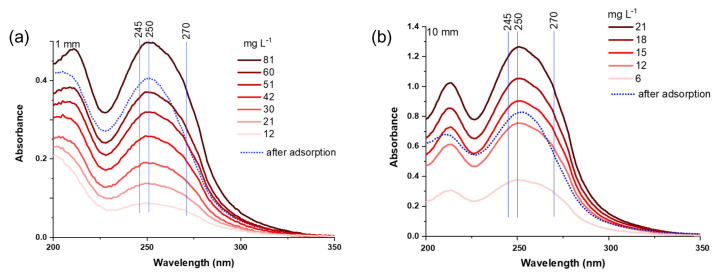
UV spectra of NN mixtures at different concentrations and spectra of the supernatant from a suspension with an initial NN concentration of (**a**) 300 mg L^−1^ or (**b**) 240 mg L^−1^, after 24 h of contact time with HY adsorbent (dotted lines).

In Figure 7a,b, the spectra of supernatants after the adsorption of solutions with total NN concentrations (with equal concentrations of individual NNs) of 300 and 240 mg L^−1^, respectively, are compared with the spectra of NNs at different concentrations. As can be observed, when all three NNs are present in the solution, their adsorption is evident to a significant extent. However, some inconsistencies in the degree of adsorption of individual NNs can be noted. Specifically, the intensity of composite bands on the side of longer wavelengths decreases more rapidly, where the absorption maximum of IC is located, affecting the perceived adsorption capacity for IC.

In Table 2, the adsorbed amounts of each NN in the mixture are determined from the spectra of the supernatants after adsorption using a system of Equations (1)–(3) and HPLC results. At lower initial concentrations, the adsorbed amounts of individual NNs are comparable. When the initial concentrations of all three NNs reach 100 mg L^−1^, a significant lag in the adsorption of TM is evident. When the number of available active sites is close to the saturation limit, competition occurs among the individual NNs for the few remaining sites on the adsorbent. The calculated adsorption energies support this lower adsorption affinity for TM.

### 2.4. Thermal Stability of Adsorbed Pesticides

Understanding the thermal properties of adsorbates is fundamental for adsorbent regeneration, as it provides insight into their stability, desorption behavior, and potential decomposition during the regeneration process, which was discussed in detail for AA [10]. Each adsorption step after the regeneration of thermally stable zeolite support enables additional pesticide retention with a minor loss of capacity for IC and AA [24].

**Figure 8:**
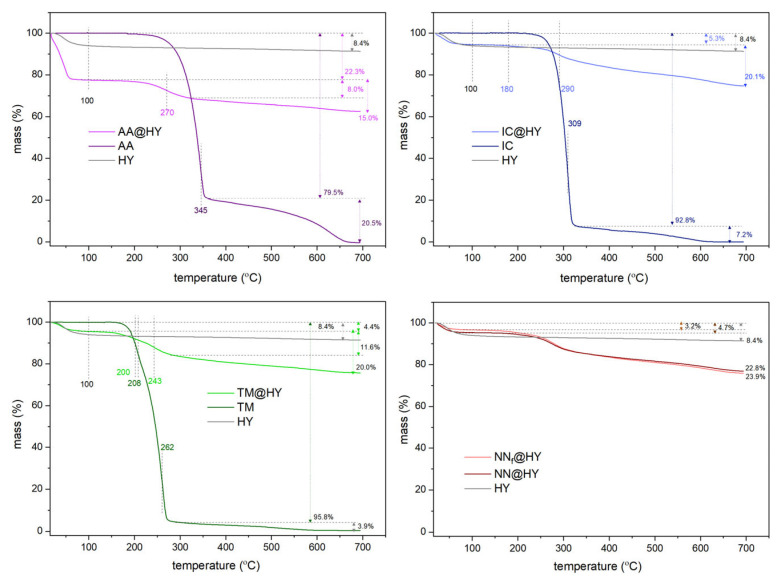
TG profiles of pristine HY, NNs, and NN-saturated samples, for individual components and mixture.

The thermogravimetric (TG) mass loss profiles for the three pesticides are similar, as presented in Figure 8. The thermogram of AA aligns with previously published results on its thermal behavior in an oxygen atmosphere [10]. Water desorption at around 100 °C is followed by a two-step mass loss observed at approximately 345 °C and 600 °C. After decomposition occurs with a mass loss of about 79%, the second step leads to AA’s complete decomposition to gaseous products. The presence of zeolite exhibits a catalytic effect, as evidenced by a shift in the decomposition temperature to 270 °C. This temperature is ascribed to the formation of carbonized material, as seen in Raman spectra [10]. Moreover, after 50 days, the TG curve of AA@HY drops an additional 0.5% in the mass loss region ascribed to AA (100–700 °C).

The HY zeolite contains less than 10% moisture, most of which is lost by 100 °C. A slightly higher moisture content of 22.2% is detected within the AA@HY sample. The mass loss of 15.5% corresponds to the adsorbed AA amount on the zeolite. Calculated as the mass ratio, this result corresponds to 183 mg g^−1^ and is consistent with findings from adsorption measurements using spectrometric and chromatographic data.

The TG profiles of TM and IC are similar, both displaying distinct decomposition steps. For IC, an additional mass loss step is observed at 180 °C alongside one at 290 °C, which is shifted compared to the decomposition step of pure IC at 309 °C. The adsorbed amount of IC accounts for approximately 20.1% of the composite mass. A comparable adsorption value is observed for TM (20%), with slightly less pronounced temperature shifts. For pure TM, mass loss occurs at 208 °C and 262 °C, while for TM@HY, these transitions shift to 200 °C and 243 °C. TM@HY and IC@HY retain a lower amount of physisorbed water due to a lower surface polarity of TM and IC compared to AA adsorbed on HY. This feature led to slightly higher adsorbed amounts calculated from TG results for IC and TM (ca. 250 mg g^−1^) than witnessed from spectrometric/HPLC findings.

When comparing the TG profiles of samples with the adsorbed NN mixtures, it can be observed that the differences between pure NNs and their agrochemical formulations are minimal. The HY zeolite exhibits only a 1% higher adsorption capacity for pure NNs. A similar trend was noted in batch adsorption studies, although the differences were slightly more pronounced.

The catalytic impact of zeolite on the thermal behavior of the pesticides is evident, confirming adsorption capacities tested by other methods. These findings support zeolite’s role in the efficient regeneration of spent adsorbents at lower temperatures, in line with previous results for AA adsorbent regeneration [10].

## 3. Materials and Methods

### 3.1. Materials

Insecticides acetamiprid—AA (min. 99%), imidacloprid—IC (min. 97%), and thiamethoxam—TM (98%) were supplied by Bayer AG, Leverkusen, Germany.

Commercial pesticide formulations were also used (labeled “F”), with active component concentrations verified using appropriate calibration curves for standards. Formulations of AA (Tonus, Galenika Fitofarmacija, Acetamiprid 200 g kg^−1^) and TM (Asteria, Galenika Fitofarmacija, Thiamethoxam 250 g kg^−1^) were in the form of a water-soluble powder/granules and IC in the form of a concentrated solution (Confidor SL200, Bayer, Imidacloprid 200 g L^−1^).

The zeolite HY (FAU) framework, obtained from Zeolyst International (Conshohocken, PA, USA) with Si/Al = 40 (code CBV780) and a specific surface area of 780 m^2^ g^−1^, was used as an adsorbent. Before use, HY zeolite was dried at 120 °C for 2 h and stored in a desiccator with silica gel.

### 3.2. Methods

Ultraviolet (UV) absorption spectra were recorded in a quartz cuvette with an optical path of 1 or 10 mm, in the 200–400 nm range, on an Evolution 220 UV/Vis spectrometer (Thermo Scientific, Waltham, MA, USA). The detection limit (LOD) can be calculated as 3.3 S_b_/a and the quantification limit (LOQ) as 10 S_b_/a. Here, S_b_ is the standard deviation of the intercept, and a is the slope of the calibration curve.

The FTIR spectra of samples were recorded in the mid-IR range (4000–400 cm^−1^) using a Nicolet iS20 FT-IR Spectrometer (Thermo Fisher Scientific, Waltham, MA, USA) operating in ATR mode with a monolithic diamond crystal. The spectral resolution was 4 cm^−1^, and each measurement was averaged over 32 scans.

High-performance liquid chromatography (HPLC) measurements were carried out using the Bischoff chromatography system (Bischoff Chromatography, Leonberg, Germany) consisting of an HPLC Compact Pump Model 2250, Rheodyne 7725i sample injector equipped with 20 µL sample loop, reversed-phase C18 column (150 × 4.6 mm, 5 μm particle size diameter, ProntoSIL 120-5-C18 AQ PLUS), UV-Vis Lambda 1010 detector, TG-14 Vacuum degasser, and LC-CaDI 22-14 Interface. The system was fully controlled, and chromatographic data acquisition and processing were performed by McDAcq32 software (Bischoff, Leonberg, Germany). The NN determination was performed at 245, 250, and 270 nm (for AA, IC, and TM, respectively) and 260 nm (for NNs in mixtures). The mobile phase was water/acetonitrile (70:30, *v*/*v*), and isocratic elution was performed at a 1.0 mL min^−1^ flow rate.

### 3.3. Adsorption Studies

For the investigation of the adsorption behavior of individual NNs, aqueous solutions with a concentration of 300 mg L^−1^ were employed. To accurately discriminate between distinct competitive binding mechanisms, experimental assays were conducted in triplicate at sufficiently elevated target concentrations. Consequently, lower concentrations that exhibited complete adsorption in preliminary assessments were excluded from further analysis. Targeted concentrations of NNs were tested in a rather broad range, going from 10 to 100 mg L^−1^ per constituent. The batch adsorption suspensions included 10 mg of Y zeolite and 10 mL of NN solution. The suspensions were placed in an ultrasonic bath for 60 min and were allowed to equilibrate for 24 h at 23 °C. In previous studies, we investigated the adsorption equilibration time [11,24]. Although neonicotinoids adsorb rapidly onto zeolitic materials, allowing a longer equilibration period results in less variability and more consistent results. After the equilibration period, the dispersions were filtered through a 0.45 µm Phenex nylon syringe filter (Phenomenex), and the amount of NNs in the supernatant was measured by UV/Vis and HPLC methods. The amounts of adsorbed NNs were calculated per gram of Y zeolite.

The quantitative measurement of pesticide retention in individual and mixture adsorption systems was examined through thermogravimetric (TG) analysis using an SDT 2960 thermoanalytical device (TA Instruments, New Castle, DE, USA). NN-saturated Y zeolite samples for post-adsorption FTIR and TG analyses were prepared by drying the centrifuged precipitate, obtained after the equilibration time, under ambient air and room temperature conditions. The thermal properties of the pristine and NN-saturated Y zeolites were investigated as temperature increased from ambient conditions to 700 °C at a heating rate of 10 °C min^−1^, under a synthetic air stream flowing at 70 mL min^−1^.

### 3.4. Theoretical Modeling

Theoretical modeling of molecular structures and interactions was performed using several quantum chemistry programs, with methods selected based on the trade-off between computational efficiency and the level of accuracy required at different stages of this study. Using MOPAC2016 [39], ten different conformations for each neonicotinoid were optimized at the PM7 semiempirical level. PM7 was chosen for its efficiency and reasonable accuracy in geometric optimization of organic molecules. The lowest-energy conformers were then subjected to further analysis. Self-consistent field calculations and electron density computations were then performed using the QUANTUM ESPRESSO suite [40,41,42] employing the Perdew–Burke–Ernzerhof (PBE) exchange-correlation functional within the generalized gradient approximation (GGA). Norm-conserving pseudopotentials were used, with a plane-wave kinetic energy cut-off of 50 Ry and a charge density cut-off of 500 Ry. The gamma point calculation was employed for Brillouin zone sampling, appropriate for isolated molecular systems. Each molecule was placed in a box of 25 Å × 25 Å × 25 Å. Although dispersion interactions were not explicitly included at this stage, the focus was on electron density and ESP features, for which PBE provides a reliable description. The resulting electron density and ESP files were imported into the Multiwfn program [43], which was used to generate ESP surface maps and compute the percentage distribution of ESP values.

The interaction mechanism between the Y zeolite network and NNs was analyzed further. Due to the complexity and large number of atoms, the approximate DFTB+ [44] was used instead of the DFT method. Due to the lack of slakos files for all present atomic species, the GFN2-xTB Hamiltonian was used. GFN2-xTB provides a robust parameterization that supports materials science applications, where detailed structural and energetic insights are vital. However, the application of that Hamiltonian limits the calculations of other quantities offered by the DFTB+ v23.1 software (open source) itself.

Interaction energy was calculated using the formula $\Delta E_{M/Y}=\;E_{M/Y}\;-\;\left(E_{M}+E_{Y}\right)$ where $E_{M/Y}$ is the calculated energy for the pesticide molecule within the Y framework, $E_{M}$ is the calculated energy for the pesticide molecule, and $E_{Y}$ is the calculated energy for the Y zeolite model.

## 4. Conclusions

The competitive adsorption of neonicotinoid pesticides in mixtures was investigated. A detailed analysis of the spectroscopic and chromatographic findings showed exceptionally high adsorption capacities of Y zeolite for all three neonicotinoids tested—acetamiprid, imidacloprid, and thiametoxam. When tested individually, the highest adsorption capacities were 204, 244, and 207 mg g^−1^ for AA, IC, and TM. When found in mixtures, competitive binding leads to uneven adsorption of all three pesticides at higher initial concentrations, resulting in low thiamethoxam binding due to its lower energy of adsorption. The resolution of each component’s retention is revealed through molar absorptivities and is well correlated with HPLC detection. This allows the correct quantification of each pesticide in a mixture within the 7–100 mg L^−1^ range, even if HPLC is unavailable. Additionally, adsorption from agrochemical formulations shows a decrease in retention due to the fillers. A detailed analysis of the FTIR spectra and DFT/semiempirical calculations identified the exact adsorption centers for binding individual neonicotinoids.

The most intense acetamiprid vibrations, corresponding to C≡N and C=N groups, shift to higher wavenumbers upon adsorption, indicating a strong interaction of the proton acceptor in the hydrogen bond with the zeolite surface, confirmed by charge distribution maps. On the other hand, IC acts as a proton donor, participating in hydrogen bonding through the N-H site within the imidazoline ring, interacting with oxygen atoms on the zeolite framework. Calculations suggest that oxygens in the TM end group serve as anchor points to the zeolite framework via dipole–dipole interaction. The thermal profiles of adsorbed pesticides confirmed the catalytic influence of zeolite on their degradation at lower temperatures. Additionally, TG results on adsorbed amounts of pesticides, both individual and in a mixture, align with adsorption capacities measured by other methods. The obtained results highlight zeolite’s excellent retention of neonicotinoids and efficiency in saturated adsorbent regeneration at lower temperatures. Theoretical modeling of neonicotinoids’ interaction with zeolite facilitates the design of efficient remediation systems, providing a simple and cost-effective approach for screening in polluted water tables.

## Figures and Tables

**Table 1:** Molar absorption coefficients, limits of detection, and limits of quantification of individual NNs determined by UV/Vis spectroscopy using a cuvette with a 1 mm optical path.

	AA			IC			TM		
λ (nm)	245	250	270	245	250	270	245	250	270
ε (m^2^ mol^−1^)	**2127** **± 9**	2020 ± 8	657 ± 5	598 ± 8	892 ± 6	**2265** **± 6**	1645 ± 10	**1744** **± 11**	823 ± 7
LOD (mg L^−1^)	**3**	3	4	2	2	**2**	5	**5**	5
LOQ (mg L^−1^)	**8**	8	12	5	4	**5**	13	**13**	13

**Table 2:** Adsorption capacities as determined using UV-Vis spectroscopy and HPLC.

	q (mg g^−1^)							
	AA		IC		TM		Σ	
Conc. (AA + IC + TM) mg L^−1^	HPLC	UV/Vis	HPLC	UV/Vis	HPLC	UV/Vis	HPLC	UV/Vis
10 + 10 + 10	9.4	/	8.8	/	9.6	/	27.8	/
20 + 20 + 20	19.6	/	17.9	/	19.6	/	56.1	/
40 + 40 + 40	40.6	39 ^#^	36.6	39 ^#^	39.5	39 ^#^	116.7	117
60 + 60 + 60	59.0	60 ^#^	58.2	59 ^#^	57.2	55 ^#^	174.4	174
80 + 80 + 80	79.5	(78 ^#^) 76 *	75.9	(77 ^#^) 77 *	70.4	(71 ^#^) 73 *	225.9	226
100 + 100 + 100	90.0	88 *	88.5	89 *	49.1	57 *	227.6	234

^#^ cuvette with an optical path of 10 mm; * cuvette with an optical path of 1 mm.

## Data Availability

Dataset available on request from the authors.

## Supplementary Materials

The following supporting information can be downloaded at https://www.mdpi.com/article/10.3390/molecules30153267/s1, Figure S1: (a) UV spectra of acetamiprid (AA), imidacloprid (IC), and thiamethoxam (TM) at different concentrations (30, 20, 15, 10, 7.5, 5, and 2.5 mg L^−1^); (b) calibration curves at λ_max_ (245, 270, and 250 nm), optical path: 10 mm; Table S1: Molar absorption coefficients, limits of detection, and limits of quantification of individual NNs determined by UV/Vis spectroscopy using a cuvette with a 10 mm optical path; Figure S2: Chromatograms corresponding to the initial standard mixture NN300: 1—thiamethoxam, 2—imidacloprid, 3—acetamiprid, (a) before and (b) after adsorption on HY zeolite, NN300@HY. Table S2: Retention factor, retention time, and linear curve parameters. Figure S3: UV spectra of individual NN concentrations, their mathematical sums, and spectra of multicomponent solutions. The numbers represent the concentration of NN in mg g^−1^; Figure S4: The concentrations of individual NNs in mixtures, determined using molar absorption coefficients and corresponding UV/Vis spectra.
