# Supplementary material for: Spectroscopic, Thermally Induced, and Theoretical Features of Neonicotinoids’ Competition for Adsorption Sites on Y Zeolite
Source: Molecules. 2025 Aug 4;30(15):3267. doi: 10.3390/molecules30153267 (PMC12348587; doi:10.3390/molecules30153267)
Supplement: Supplementary file 1 [file molecules-30-03267-s001.zip › molecules-3781181-supplementary.pdf]

Supporting information

Spectroscopic, Thermally Induced, and Theoretical Features of Neonicotinoids' Competition for Adsorption Sites on Y Zeolite

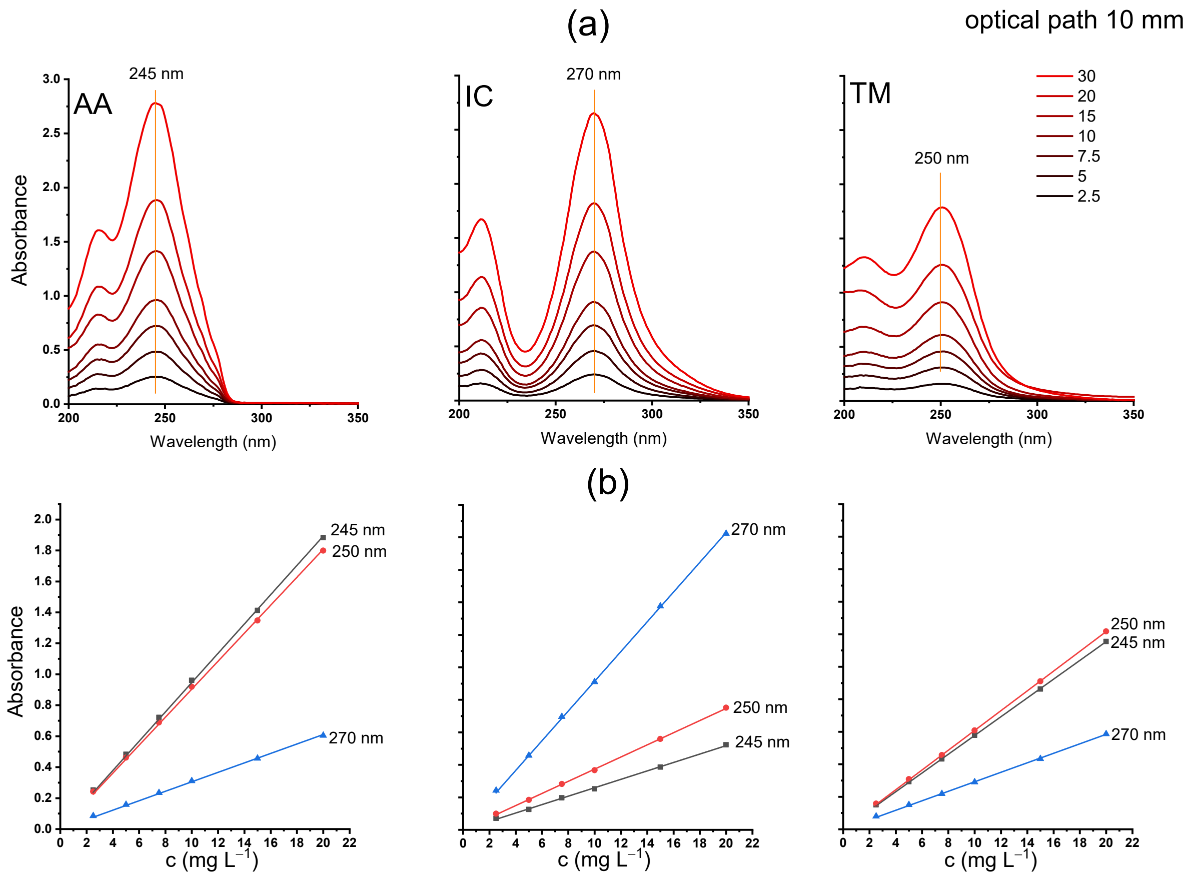

Figure S1. (a) UV spectra of acetamiprid (AA), imidacloprid (IC), and thiamethoxam (TM) at different concentrations (30, 20, 15, 10, 7.5, 5, and 2.5 mg L^{-1}); **(b)** Calibration curves at λ_{max} (245, 270, and 250 nm), optical path: 10 mm.

Table S1. Molar absorption coefficients, limits of detection, and limits of quantification of individual NNs determined by UV/Vis spectroscopy using a cuvette with a 10 mm optical path.

	AA			IC			TM		
λ (nm)	**245**	250	270	245	250	**270**	245	**250**	270
ε (m^2 mol^{-1})	**2108 ±10**	**2013 ±10**	**680 ± 5**	**662 ± 5**	**955 ± 4**	**2338 ± 7**	**1672 ± 4**	**1774 ± 3**	**852 ± 4**
LOD (mg L^{-1})	**0.2**	0.2	0.2	0.6	0.4	**0.1**	0.2	**0.1**	0.4
LOQ (mg L^{-1})	**0.4**	0.6	0.5	2.0	1.0	**0.2**	0.4	**0.4**	2.0

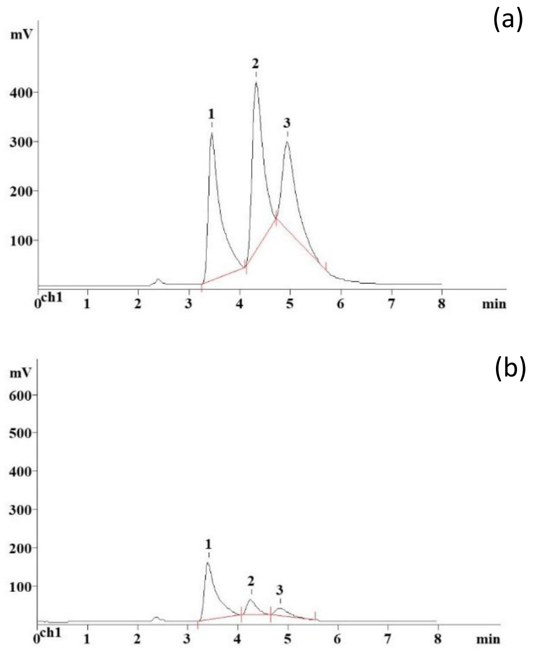

Figure S2. Chromatograms corresponding to the initial standard mixture NN300: 1 – thiamethoxam, 2 – imidacloprid, 3 – acetamiprid, **(a)** before and **(b)** after adsorption on HY zeolite, NN300@HY.

Table S2. Retention factor, retention time, and linear curve parameters.

Component name	Retention factor (RF)	Retention time (t_R)/min	linear curve parameters
**Thiamethoxam**	0.4103	3.423	Q=0.410·A-6.221<br>RSD=1.031% R=0.9999
**Imidacloprid**	0.4004	4.256	Q=0.400·A+36.376<br>RSD=3.126% R=0.9991
**Acetamiprid**	0.575	5.101	Q=0.575·A-17.720<br>RSD=1.653% R=0.9998

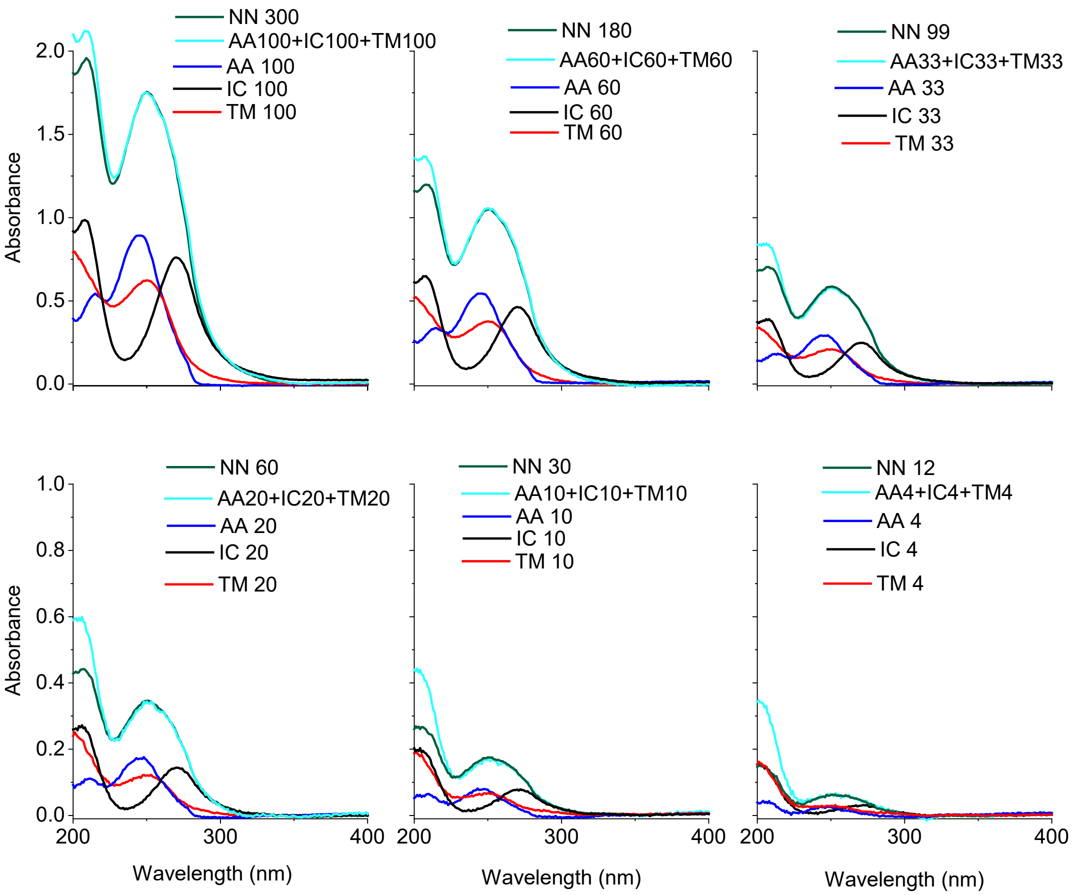

Figure S3. UV spectra of individual NN concentrations, their mathematical sums, and spectra of multicomponent solutions. The numbers represent the concentration of NN in mg g^{-1}.

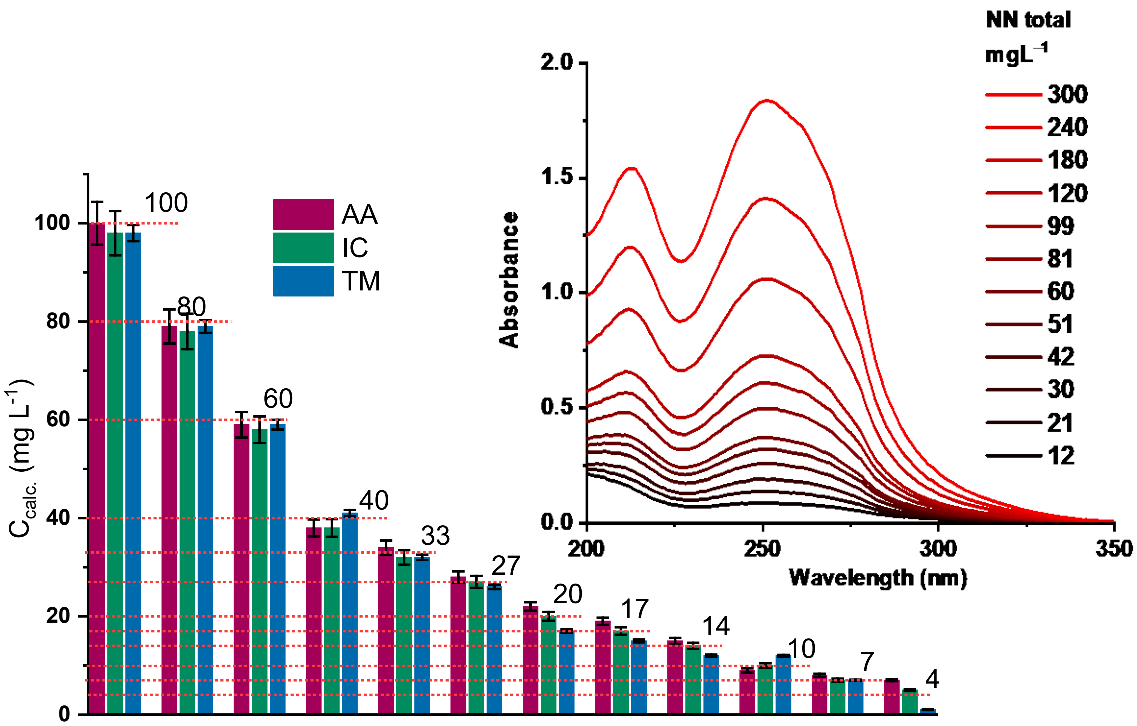

Figure S4. The concentrations of individual NNs in mixtures, determined using molar absorption coefficients and corresponding UV/Vis spectra.
